# 

**Published:** 1891-02-21

**Authors:** 


**Where Do We Stand?** . . . . . . . . 307

**Passing Topics.**—The Hospital Saturday Fund Disagreement 308

**The Mark of the Beast.**—Some Notes on Criminal Anthropology . . . . . . . . 309

**Everybody's Page** . . . . . . . . 310

**The Microscope in Medicine.** By FRANK J. WETHERED, M.D.—VI. Mounting Specimens . . . . . . 311

**Notes and News** . . . . . . . . 312

**General Charities—Scraps and Gleanings** . . . 313

**Annotations.**—What is Madness?—Dr. Rentoul is Sarcastic—Threatened Closing of a Hospital Ward . . . . 314

**The Lords' Committee** . . . . . . . . 318

**The Practitioner's Mirror and Retrospect:—**

MEDICINE.—Some Functional Disorders of the Heart, 315; Some Medical Aspects of Tropical Colonisation . . 316

SURGERY.—Chronic Disease of the Knee Joint in Children . 317

TOXICOLOGY—Poisoning by Antipyrin . . . . . 317

THE PRACTITIONER'S BOOKSHELF—Dental Chemistry and Metallurgy . . . . . . . . 317

NEW DRUGS, APPLIANCES, AND THINGS MEDICAL.—Shepherd's "Warmers"—Caffyn's Liquor Carnis—Bailey's Concentrated Clam Juice . . . . . . 318

**The Student of Medicine.**—Appointments—Vacancies—Pass Lists—Athletics.

### EXTRA SUPPLEMENT—THE NURSING MIRROR.

**En Passant.**—Southend Institute—Out of Trouble—Leeds District Nurses—Workhouse Infirmaries—Canterbury Institute—Infirmary Matters—Short Items—A New Home . cxiii

**Lectures on Surgical Ward Work and Nursing.** By ALEX. MILES, M.B. Edin., C.M., F.R.C.S.E. Lecture XIV.—Ligatures and Tourniquets . . . . . cxiv

**Nursing Medals and Certificates.**—The Suakin Medal cxv

**For Reading to the Sick.**—Energy. **Appointment** cxv

**American News** . . . . . . . . cxvi

**The Nurses' Bookshelf.**—Physiology and Hygiene for Home Nursing—The Dignity of Woman's Health, &c. . cxvi

**Everybody's Opinion.**—The Dinner Hour . . . . cxvii

**Princess Christian's Daughter** . . . . . cxvii

**Presentations.—Notes and Queries** . . . . cxvii

**Off Duty.**—Nurse Hilary (*continued*) . . . . cxviii

**Amusements and Relaxation** . . . . . vcx ii

